# Differential Effects of Chronic Pulsatile versus Chronic Constant Maternal Hyperglycemia on Fetal Pancreatic **β**-Cells

**DOI:** 10.1155/2012/812094

**Published:** 2012-10-22

**Authors:** Mackenzie S. Frost, Aqib H. Zehri, Sean W. Limesand, William W. Hay, Paul J. Rozance

**Affiliations:** ^1^Department of Pediatrics, Drexel University College of Medicine, Philadelphia, PA, USA; ^2^Department of Animal Sciences, University of Arizona, Tucson, AZ 85719, USA; ^3^Department of Pediatrics, UCD Perinatal Research Center, University of Colorado School of Medicine, 13243 East 23rd Avenue, MS F441, Aurora, CO 80045, USA

## Abstract

Constant maternal hyperglycemia limits, while pulsatile maternal hyperglycemia may enhance, fetal glucose-stimulated insulin secretion (GSIS) in sheep. However, the impact of such different patterns of hyperglycemia on the development of the fetal **β**-cell is unknown. We measured the impact of one week of chronic constant hyperglycemia (CHG, *n* = 6) versus pulsatile hyperglycemia (PHG, *n* = 5) versus controls (*n* = 7) on the percentage of the fetal pancreas staining for insulin (**β**-cell area), mitotic and apoptotic indices and size of fetal **β**-cells, and fetal insulin secretion in sheep. Baseline insulin concentrations were higher in CHG fetuses (*P* < 0.05) compared to controls and PHG. GSIS was lower in the CHG group (*P* < 0.005) compared to controls and PHG. PHG **β**-cell area was increased 50% (*P* < 0.05) compared to controls and CHG. CHG **β**-cell apoptosis was increased over 400% (*P* < 0.05) compared to controls and PHG. These results indicate that late gestation constant maternal hyperglycemia leads to significant **β**-cell toxicity (increased apoptosis and decreased GSIS). Furthermore, pulsatile maternal hyperglycemia increases pancreatic **β**-cell area but did not increase GSIS, indicating decreased **β**-cell responsiveness. These findings demonstrate differential effects that the pattern of maternal hyperglycemia has on fetal pancreatic **β**-cell development, which might contribute to later life limitation in insulin secretion.

## 1. Introduction

Fetuses exposed to chronic hyperglycemia secondary to maternal diabetes are prone to develop *β*-cell hyperplasia and increased insulin secretion that underlie increased postnatal risk of exaggerated glucose-stimulated insulin secretion (GSIS) and hyperinsulinemic hypoglycemia [1–6]. In contrast, severe maternal diabetes during pregnancy that is complicated by poor glycemic control and vasculopathy and is further associated with intrauterine growth restriction (IUGR) results in decreased fetal pancreatic *β*-cell area and GSIS [3]. In both cases, not only are there short-term complications for these offspring, but also long-term consequences. These long-term consequences for the offspring include an increased risk of developing adult onset diabetes [3].

Previous studies in pregnant sheep have tested the impact of chronic hyperglycemia on fetal *β*-cell function, using controlled experimental manipulation of maternal and fetal glucose concentrations and in vivo measurement of fetal insulin secretion to begin to determine mechanisms responsible for the impact of different patterns of maternal and fetal glucose concentration, as occur in diabetic pregnancies, on fetal GSIS [7–11]. Such studies demonstrated that constant maternal hyperglycemia produced an initial increase in fetal glucose and insulin concentrations [9], but over eight to ten days of constant high maternal and fetal glucose concentrations the fetal insulin concentrations returned to normal [8, 9]. Furthermore, after ten days of constant maternal hyperglycemia, fetal GSIS and arginine-stimulated insulin secretion (ASIS) were decreased compared to normal fetuses [8, 9].

More variable results were obtained when chronic hyperglycemia was limited to three one-hour pulses each day (pulsatile hyperglycemia, akin to meal-associated hyperglycemia); in these studies over a similar period of 10–11 days, fetal GSIS increased [9]. However, a subsequent study demonstrated that changes in fetal GSIS following chronic pulsatile maternal hyperglycemia depended on the degree and duration of the increased glucose concentrations [12]. 

Despite such striking differences in fetal GSIS in response to different patterns of maternal and fetal glucose concentrations over extended periods, these studies did not provide insight into mechanisms responsible for such unique changes in fetal pancreatic function. Specifically, there has not been any morphological evaluation of the fetal pancreas in response to such different hyperglycemic patterns. It is not known, for example, whether chronic constant maternal hyperglycemia, even confined to the latter portion of gestation, will lead to or directly produce other signs of fetal *β*-cell toxicity besides decreased GSIS, such as increased *β*-cell apoptosis or decreased pancreatic *β*-cell area. It also is not known how the variability in fetal GSIS following chronic pulsatile maternal hyperglycemia relates to fetal *β*-cell area, apoptosis, mitosis, or size. 

Therefore, we undertook the studies reported herein to test whether chronic constant fetal hyperglycemia for seven days would decrease fetal pancreatic *β*-cell area in association with decreased *β*-cell mitosis and increased apoptosis. We also tested whether chronic pulsatile maternal hyperglycemia (limited to seven days) would result in increased *β*-cell area due to increased *β*-cell mitosis and decreased apoptosis. 

## 2. Materials and Methods

### 2.1. Animal Preparation

Studies were conducted in pregnant Columbia-Rambouillet sheep carrying single fetuses in compliance with the Institutional Animal Care and Use Committee, University of Colorado Denver at the Perinatal Research Center in Aurora, CO. This laboratory is accredited by the National Institutes of Health, the United States Department of Agriculture, and the American Association for Accreditation of Laboratory Animal Care. Sheep underwent surgery at 118–122 days gestation (term = 148 days). Maternal catheters were placed into the femoral artery and vein via a groin incision. Fetal catheters were placed into the abdominal aorta and inferior vena cava via pedal incisions as previously described [13]. Fetal and maternal catheters were flushed daily with 1.5 mL (fetus) or 3 mL (maternal) heparinized saline solution (50 U/mL heparin in 0.9% NaCl in H_2_0). Animals were allowed a minimum of three recovery days prior to initiation of experimental infusions.

### 2.2. Experimental Protocol

Animals were randomly assigned to one of three study groups and managed as previously described: (1) euglycemia with saline infusion (control, *n* = 7); (2) continuous 70% dextrose (w/v, D70) infusion adjusted to increase maternal arterial plasma glucose concentrations 80% based on an average of twice daily maternal arterial plasma glucose concentration measurements (constant hyperglycemia, CHG, *n* = 6); (3) basal D70 infusion at a rate adjusted to increase maternal plasma glucose 20% with additional 60 minute infusions of D70 three times a day (8 AM, 2 PM, and 8 PM) at a rate targeted to increase maternal arterial plasma glucose concentrations 80% higher than controls (pulsatile hyperglycemia, PHG, *n* = 5) [9]. These treatments were maintained for seven days. Maternal glucose concentrations were checked at least twice daily, with additional measurements in the PHG group. On day seven of the infusion period maternal and fetal arterial blood acid-base balance, oxygen (PaO_2_, SaO_2_, and arterial blood O_2_ content), and carbon dioxide (PaCO_2_) were measured. At baseline and throughout the infusion fetal arterial plasma glucose and insulin concentrations were measured. 

### 2.3. In Vivo Fetal Insulin Secretion

On day seven of the maternal infusions GSIS was measured with a square wave fetal hyperglycemic clamp followed by an arginine bolus to measure glucose potentiated ASIS [13]. Baseline arterial blood samples for fetal arterial plasma glucose and insulin concentrations were drawn at −25, −15, and −5 minutes (relative to initiation of the glucose clamp at minute 0). Fetal arterial blood also was sampled at −25 and −15 for acid-base balance, PaO_2_, SaO_2_, arterial blood O_2_ content, and PaCO_2_ measurements. The fetal hyperglycemic clamp was performed with a direct fetal glucose infusion adjusted to double fetal arterial plasma glucose concentrations [9, 13]. At 125 minutes an infusion of arginine (261 mg in 5 mL) was administered over four minutes to measure ASIS. Blood samples were drawn at 5, 10, 20, 30, 60, 90, 120, 130, 135, 145, and 155 minutes to measure fetal arterial plasma glucose and insulin concentrations. Blood was sampled at minute 60, 90, and 120 for acid-base balance, PaO_2_, SaO_2_, arterial blood O_2_ content, and PaCO_2_ measurements. After minute 155 the fetal glucose infusion was stopped and the maternal glucose or saline infusions continued overnight to allow the fetus to return to pre-GSIS study conditions.

### 2.4. Biochemical Analysis

Whole blood was collected in EDTA-coated syringes and immediately centrifuged (14,000 g) for 3 min at 4°C. Plasma was removed and the glucose and lactate concentrations were immediately determined using the YSI model 2700 select biochemistry analyzer (Yellow Springs Instruments, Yellow Springs, OH) [13]. The remainder of the plasma was stored at −70°C for insulin measurements which was by ELISA (Alpco; inter- and intra-assay CV's: 2.9 and 5.6%) [13]. For O_2_, CO_2_, pH, and hematocrit concentrations whole blood was collected in heparinized syringes, and concentrations were immediately determined using an ABL 520 analyzer (Radiometer, Copenhagen, Denmark). Oxygen content of the blood was calculated by the ABL 520 analyzer [13].

### 2.5. Organ Isolation

Necropsies and organ isolation were performed the day after measurement of in vivo insulin secretion as previously described [13]. The splenic portion of the pancreas was fixed overnight in 4% Paraformaldehyde (w/v) in Phosphate-buffered Saline (PBS), and then transferred to 70% ethanol (v/v) until it was paraffin embedded. The method by which we obtained the pancreas precluded measurement of pancreatic weight. 

### 2.6. Fetal Pancreatic Histology

Paraffin embedded tissue sections (5 *μ*m) were cut at 100 *μ*m intervals from the splenic portion of the pancreas. *β*-cell area, apoptosis, and mitosis were determined as previously described with slight modification in the dewaxing and antigen retrieval steps for sections evaluated for *β*-cell area only [14–16]. The modifications were that dewaxing was performed by washing slides twice in EZ Dewax tissue deparaffinization solution (Biogenex) for five minutes. Slides were then placed in water to rinse for five minutes followed by washing in Supersensitive Wash Buffer (Biogenex) for 5 minutes. Antigen unmasking was performed by placing the slides in Antigen Unmasking Solution (Vector) and heating to 95°C for 20 minutes. This was followed by blocking with 1.5% normal donkey serum in PBS (v/v) for 30 min. Mature endocrine hormone^+^ cells were identified with the following primary antibodies diluted in blocking buffer: guinea pig anti-porcine insulin (Dako, Carpinteria CA, 1 : 500) or mouse anti-human insulin (Abcam, Cambridge UK, 1 : 1000), mouse monoclonal anti-human glucagon, (Sigma-Aldrich, St. Louis MO, 1 : 500), rabbit anti-human somatostatin, (Dako, 1 : 500), rabbit anti-human pancreatic polypeptide, (Dako, 1 : 500). Sections were incubated at 4°C overnight and immunocomplexes detected the next day with affinigy purified secondary antiserum conjugated to Rhodamine Red (Jackson ImmunoResearch Laboratories, West Grove PA), 7-amino-4methylcoumarin-3-acteic acid (AMCA, Jackson ImmunoResearch Laboratories), and AlexaFluor 488 (Molecular Probes, Eugene OR) [14]. *β*-cell mitosis and apoptosis were determined as the percentage of insulin^+^ cells which were also positive for phosphorylated Histone H3 or terminal deoxynucleotidyl transferate (TdT)-mediated dUTP nick translation end labeling (TUNEL), respectively, as previously described [14]. *β*-cell size was determined by dividing the insulin^+^ area by number of nuclei within that area as determined with 4′,6 diamidino-2-phenylindole (DAPI, Vector Laboratories, Burlingame CA) [14].

### 2.7. Statistical Analysis

Statistical analysis was performed using SAS version 9.1 or GraphPad Prism 4.0 for Windows. Results are expressed as mean ± SEM. A mixed models ANOVA with a random animal term to account for repeated measurements made within an animal was performed to determine effects of treatment group (control, CHG, or PHG), time (days of treatment or minutes of hyperglycemic glucose clamp), and treatment-time interactions for all in vivo measurements except for those which were only measured at the end of the glucose clamp infusions. Measurements made only once, including fetal weight, fetal length, and pancreatic *β*-cell area, size, mitosis, and apoptosis, were compared using a one way ANOVA or the Kruskal-Wallis test.

## 3. Results

### 3.1. Maternal Parameters during Treatment

**Figure 1:**
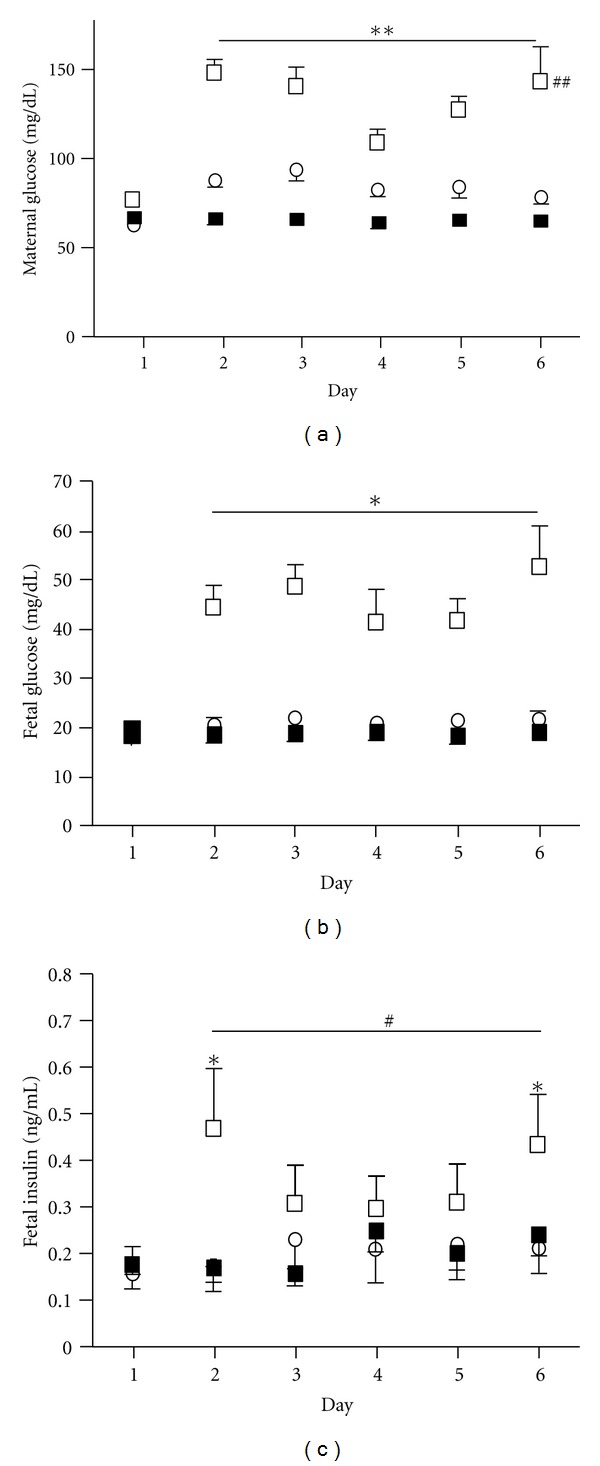
Maternal and fetal glucose and fetal insulin concentrations. Maternal (a) and fetal (b) arterial plasma glucose and fetal arterial plasma insulin (c) concentrations were measured throughout the infusion in CHG (*n* = 6, □), PHG (*n* = 5, ○), and control animals (*n* = 7, ■). ∗∗ indicates a significant difference between both CHG and PHG from controls for maternal glucose concentrations, *P* < 0.0001. ## indicates a significant difference between CHG and PHG animals for maternal glucose concentrations, <0.0001. ∗ indicates a significant difference between CHG and both PHG and control groups for fetal arterial glucose and insulin concentrations, *P* = 0.026. # indicates a significant increase in CHG fetuses only compared to their baseline (Day 1) for fetal arterial insulin concentrations, *P* = 0.028.

The infusion rate of D70 in the CHG group started at 13.2 ± 1.1 gm/hr and increased significantly over the seven day study period (*P* = 0.0003). The maximum rate was on day five, 19.3 ± 1.2 gm/hr, at which point the rate decreased slightly to a final rate of 17.9 ± 2.2 gm/hr. The chronic infusion rate in the PHG group did not change over time and averaged 1.1 ± 0.1 gm/hr. The rate of the one hour bolus infusions also did not change over time and averaged 14.7 ± 1.4 gm/hr. Maternal arterial plasma glucose concentrations were significantly increased in the CHG and PHG groups compared to controls throughout the infusion (*P* < 0.0001, Figure 1(a)). Furthermore, arterial plasma glucose concentrations in the CHG group were significantly increased compared to the PHG group (*P* < 0.0001, Figure 1(a)). To produce “pulsatile” hyperglycemia and model meal associated hyperglycemia in pregnant women, the PHG group received a 60 minute dextrose infusion that increased their arterial plasma glucose concentrations to an average of 114 ± 3 mg/dL three times a day. There was a slight, but statistically significant, increase in arterial pH in both the CHG and PHG groups compared to controls (*P* ≤ 0.028, Supplementary Table available online at doi:10.1155/2012/812094), but no other changes were noted in maternal acid-base balance, O_2_ values (PaO_2_, SaO_2_, and arterial blood O_2_ content), or PaCO_2_.

### 3.2. Fetal Parameters during Treatment

Fetal arterial plasma glucose concentrations were significantly increased in the CHG group compared to both control and PHG fetuses throughout the maternal dextrose infusion period (*P* < 0.0001, Figure 1(b)). There were no differences in fetal arterial plasma glucose concentrations between PHG and control groups. Fetal arterial plasma insulin concentrations did not change in the control and PHG groups (Figure 1(c)). In the CHG group insulin concentrations increased on day two and remained increased throughout the infusion compared to day one (baseline), (*P* ≤ 0.028, Figure 1(c)). There were small but statistically significant changes in the fetal blood pH, PaCO_2_, and lactate concentrations (Supplementary Table).

### 3.3. Fetal Insulin Secretion

**Figure 2:**
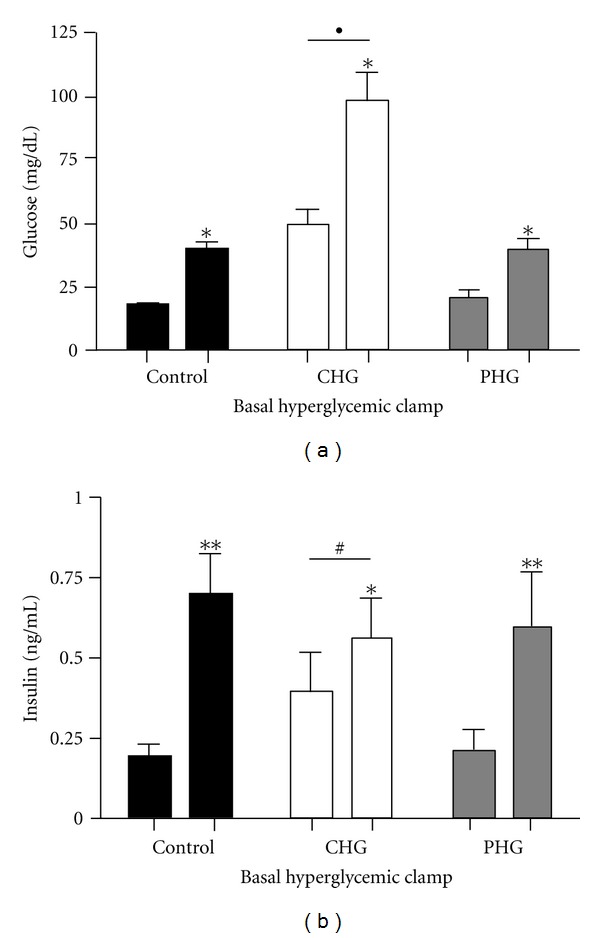
Fetal glucose-stimulated insulin secretion. A square-wave fetal hyperglycemic clamp was used to test insulin secretion. The average basal (minute −25, −15, −5) and hyperglycemic clamp (minute 60, 90, 120) glucose (a) and insulin concentrations (b) in control (*n* = 7, black bars), CHG (*n* = 6, white bars), and PHG (*n* = 5, gray bars) fetuses are shown. ∗, ∗∗ indicate a significant difference between basal and hyperglycemic clamp concentrations within a treatment group, *P* = 0.029, *P* < 0.0001, respectively. ● indicates a significant increase in the incremental change of glucose concentrations in CHG compared to both PHG and controls, *P* < 0.0001. # indicates a significant decrease in the incremental change of insulin concentrations in CHG compared to both PHG and controls, *P* = 0.0002.

Fetal insulin secretion was measured with a square-wave fetal hyperglycemic clamp on day seven. By design, fetal glucose concentrations were doubled in each group. This led to greater absolute increases in fetal glucose concentrations in the CHG group compared to the other groups (*P* < 0.0001, Figure 2(a)). For all groups, fetal arterial plasma glucose concentrations were significantly greater than baseline beginning at minute five and lasting throughout the hyperglycemic clamp period (*P* ≤ 0.005, Supplementary Figure). Fetal arterial plasma insulin concentrations increased in all three groups during the hyperglycemic clamps (Figure 2(b)). However, insulin concentrations in the CHG group did not become statistically greater than baseline concentrations until minute 90 of the hyperglycemic clamp (Supplementary Figure), despite significantly higher plasma glucose concentrations during the hyperglycemic clamp and a significantly greater increase in glucose concentrations between the basal and hyperglycemic clamp period. Basal insulin concentrations in the CHG fetuses were higher than in the PHG and Control groups. Insulin secretion in the CHG group, determined as the difference between the mean basal and clamp period insulin concentrations, was less than in the other groups during the hyperglycemic clamp (*P* = 0.0002, Figure 2(b)). Nevertheless, mean insulin concentrations during the hyperglycemic clamps were not different among the three groups. Insulin secretion in PHG fetuses was not different from control fetuses. Arginine-stimulated insulin secretion was not statistically different among the three groups. All groups had maximum insulin concentrations at five minutes post-arginine bolus (1.68 ± 0.24 ng/mL control, 2.61 ± 0.59 ng/mL CHG, 3.51 ± 1.94 ng/mL PHG). Although fetal acid-base balance, blood oxygen values (PaO_2_, SaO_2_, and arterial blood O_2_ content), and PaCO_2_ changed in all groups during the hyperglycemic clamp, differences among the groups were minimal (Supplementary Table).

### 3.4. Fetal Measurements, Organ Weights, and Histology of the Fetal Pancreas

**Figure 3:**
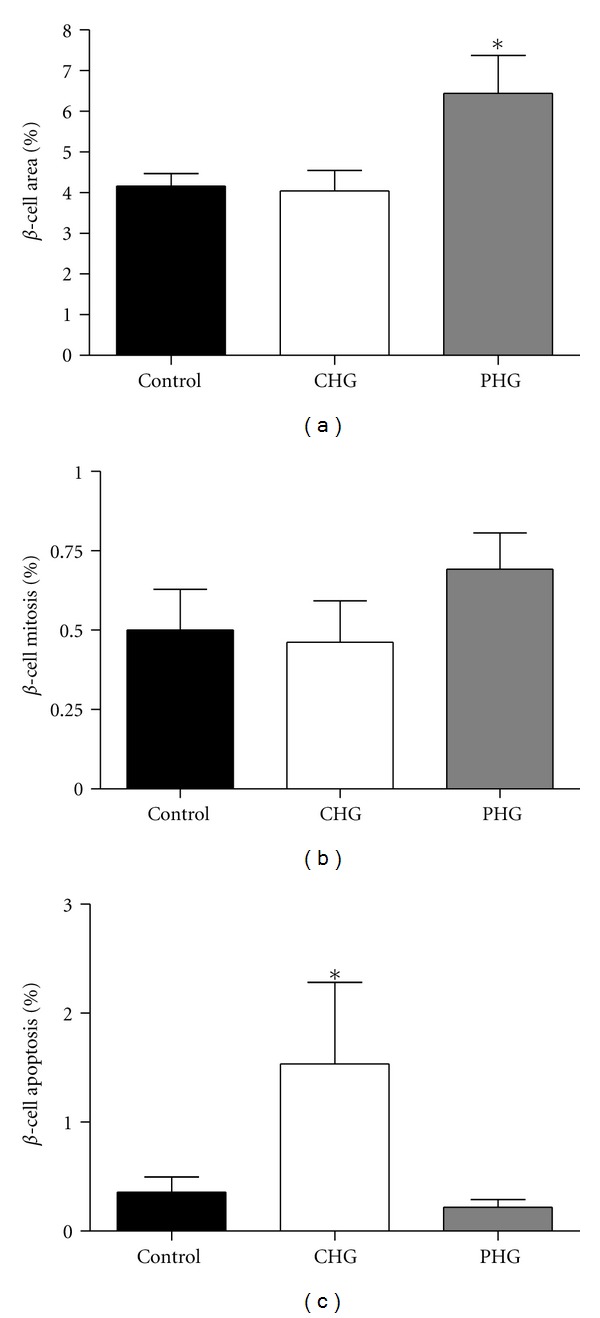
Fetal pancreatic *β*-cell area, mitosis, and apoptosis. Fetal pancreatic *β*-cell area (a), mitosis (b), and apoptosis (c) were measured in control (*n* = 7, black bars), CHG (*n* = 6, white bars), and PHG (*n* = 5, gray bars) fetal pancreases. ∗ indicates a significant difference, *P* = 0.021.

Fetal measurements and organ weights were not different among the groups (Table 1). There was no difference in the *β*-cell area between control and CHG fetal pancreases, but there was a significant increase in the *β*-cell area of the PHG pancreases (*P* = 0.021, Figure 3(a)). *β*-cell mitosis was not different among the groups (Figure 3(b)), but apoptosis was significantly increased in the CHG group (*P* = 0.021, Figure 3(c)). *β*-cell size (57.4 ± 3.5 *μ*m^2^ control, 59.3 ± 4.4 *μ*m^2^ CHG, 53.4 ± 0.8 *μ*m^2^ PHG) was not different among groups.

## 4. Discussion

The pattern of chronic maternal and thus fetal hyperglycemia is fundamental for determining the regulation of fetal *β*-cell development and function throughout the second half of gestation during which such development normally matures. In order to determine the differential effects of chronic constant versus pulsatile hyperglycemia, aimed to mimic patterns of glucose concentration common to pregnant women with diabetes, on the morphological and functional development of fetal pancreatic *β*-cells, we infused dextrose to produce different patterns of maternal hyperglycemia over one week in late gestation in pregnant sheep. There are several important novel findings of this study. First, chronic constant maternal hyperglycemia decreased fetal GSIS and increased fetal *β*-cell apoptosis, even though fetal *β*-cell size, the degree of fetal *β*-cell mitosis, and the proportional fetal *β*-cell area within the pancreas were not decreased. These unique and important observations indicate that apoptosis and decreased GSIS are early manifestations of fetal *β*-cell glucotoxicity and that decreased fetal *β*-cell glucose responsiveness is independent of decreased *β*-cell area and precedes the decrease in basal insulin secretion previously observed following ten days of chronic constant maternal hyperglycemia [8, 9]. Second, chronic pulsatile hyperglycemia increased the fetal pancreatic *β*-cell area, indicating that this cellular change might underlie increased GSIS that has been seen in other studies of chronic pulsatile maternal hyperglycemia [9]. However, because we did not find increased GSIS in the PHG fetuses despite the increase in *β*-cell area, the current results demonstrate a degree of *β*-cell dysfunction in this PHG group that is consistent with decreased GSIS seen in more recent studies of chronic pulsatile maternal hyperglycemia of a longer duration [12]. These unique observations, therefore, demonstrate cellular morphological and developmental changes that precede different patterns of insulin secretion that can be produced by pulsatile versus constant hyperglycemia, providing new insight into the pathogenesis of abnormal patterns of fetal insulin secretion that can occur in type 1 and type 2 human diabetic pregnancies.

In our studies, chronic constant maternal hyperglycemia (CHG group) for one week increased basal fetal insulin concentrations. These data are consistent with previous studies which show that over a one week period, fetal insulin concentrations initially increase with constant fetal hyperglycemia [7, 9, 11]. This is followed by a variable but progressive decline in insulin concentrations. In some fetuses insulin concentrations returned to baseline by day six to seven and for others this return to baseline required several more days [9]. More prolonged constant fetal hyperglycemia ultimately results in a decline in basal fetal insulin concentrations to even lower values than those seen in normal fetuses [8, 9].

Also consistent with previous studies, we found that chronic constant maternal hyperglycemia for seven days decreased fetal GSIS. We have extended these observations by demonstrating that the pancreatic *β*-cell area and individual *β*-cell size are unchanged. These results show for the first time that decreased *β*-cell glucose responsiveness is a mechanism for decreased fetal GSIS following chronic constant maternal hyperglycemia. We also have shown increased *β*-cell apoptosis, another indicator of fetal *β*-cell glucotoxicity. Future experiments will determine if a longer exposure to CHG also reduces *β*-cell area, which would be expected given increased *β*-cell apoptosis in this model, and would help explain decreased basal insulin concentrations following longer durations of CHG [8, 9].

An alternative explanation for decreased GSIS in the CHG fetuses is that there may be a plateau insulin concentration, representing a plateau in fetal insulin secretion and/or a balance between insulin secretion and insulin clearance, which cannot be exceeded regardless of further increases in fetal glucose concentrations, as recently demonstrated in normal fetuses [15]. However, chronic constant fetal hyperglycemia for longer than eight days results in an unequivocal decrease in GSIS and ASIS, not explainable by plateau glucose-stimulated insulin concentrations [8, 9]. These results from longer duration experiments, as well as the increased *β*-cell apoptosis demonstrated in the current study, strongly support toxicity of the fetal *β*-cell following constant maternal hyperglycemia of only seven days duration.

In the PHG fetuses we found an increase in the pancreatic *β*-cell area but not increased GSIS, as was previously observed after ten days [9]. Besides differences in the duration of chronic hyperglycemia between the current and previous study, there were differences in the fetal glucose concentrations between studies. In the current study glucose concentrations were increased by approximately 15%, which was not statistically significant, despite a significant 20% increase in maternal plasma arterial glucose concentrations. In the previous study fetal plasma arterial glucose concentrations were increased approximately 20–25% [9]. Fetal insulin concentrations also were higher in the previous study [9]. Perhaps a more important difference is the magnitude of the pulsatile hyperglycemia, which in the current study was 77% greater than controls in contrast to a 60% greater concentration in the previous study [9]. While such differences are small, a recent study tested the impact of the magnitude of the hyperglycemic pulse on GSIS and found that higher pulsatile glucose concentrations resulted in inhibition of fetal GSIS [12]. Normal GSIS in the current PHG fetuses, despite their increased *β*-cell area, indicates a degree of *β*-cell dysfunction, although one not as severe as in the CHG fetuses. 

Despite an increase in *β*-cell area in the PHG fetuses, we did not observe changes in *β*-cell size, mitosis, or apoptosis. It is possible that increased mitosis or decreased apoptosis may have been present before we obtained the pancreas as possible explanations for these observations. Furthermore, the variability in rates of *β*-cell mitosis may have precluded demonstrating a statistically significant higher rate in the PHG fetuses despite a 40% higher mean rate compared to control fetuses. We also did not measure the rate of *β*-cell neogenesis, which if increased, despite being very low near term, would tend to increase the *β*-cell population [14, 16]. Future studies will be required to obtain pancreatic tissue from PHG animals earlier in the hyperglycemic infusion period to determine the relative contributions of decreased apoptosis, increased mitosis, or neogenesis, and to determine how these processes directly and mechanistically affect fetal *β*-cell insulin secretion, at baseline and in response to glucose stimulation and stimulation by other secretagogues.

## 5. Conclusions

In summary, chronic constant hyperglycemia for seven days decreased GSIS and increased fetal *β*-cell apoptosis, though the proportional fetal *β*-cell area within the pancreas was preserved. In contrast, chronic pulsatile hyperglycemia increased the proportional fetal *β*-cell area within the pancreas. Based on these results, as well as previous studies [8, 9, 12], we speculate that differences found for human newborn GSIS following a diabetic pregnancy are due to subtle differences in fetal glucose concentrations [17–20]. Furthermore, these studies show differential effects that the pattern of maternal and fetal hyperglycemia has on fetal *β*-cells, which are plastic in their developmental and functional capacities even near the end of gestation. Such changes could allow for programming of unique *β*-cell fates that might produce later life (even lifelong) limitations in function. 

## Supplementary Material

“The supplementary material includes a table with maternal and fetal arterial blood gas and acid base data following the chronic experimental infusions. Also included in this table are the fetal blood gas and acid base data during the fetal hyperglycemic clamp. The supplementary material also includes a figure which shows time specific insulin and glucose concentrations during the fetal hyperglycemic clamp.”

## Figures and Tables

**Table 1:** Fetal Age, Weights, and Length.

	Control	Constant	Pulsatile
		Hyperglycemia	Hyperglycemia
Gestational Age (days)	134.7 ± 0.7	135.0 ± 0.6	133.6 ± 0.9
Fetal Weight (gm)	3593 ± 138	3601 ± 290	3678 ± 223
Crown Rump Length (cm)	48.8 ± 1.0	48.6 ± 0.8	50.1 ± 2.0
Liver (gm)	108.5 ± 5.2	117.9 ± 14.6	123.8 ± 6.6
Heart (gm)	29.8 ± 1.6	30.4 ± 2.4	30.7 ± 1.8
Lung (gm)	116.3 ± 4.8	100.0 ± 9.6	122.3 ± 10.3
Kidney (gm)	20.6 ± 1.5	25.5 ± 2.2	24.0 ± 1.1
Spleen (gm)	8.2 ± 0.6	7.4 ± 1.6	11.8 ± 2.2
Brain (gm)	46.4 ± 0.8	43.2 ± 0.8	44.2 ± 0.9
Carcass (gm)	2896 ± 92	2841 ± 245	2873 ± 167
Sex (% Female)	43	50	40
