# Supplementary material for: Differential Effects of Chronic Pulsatile versus Chronic Constant Maternal Hyperglycemia on Fetal Pancreatic β-Cells
Source: J Pregnancy. 2012 Oct 22;2012:812094. doi: 10.1155/2012/812094 (PMC3486011; doi:10.1155/2012/812094)

| **SUPPLEMENTARY DATA**  **Supplementary Table. Maternal and Fetal Arterial Blood Gas and Acid Base Balance Following Chronic Interventions** | | | | | | | |
| --- | --- | --- | --- | --- | --- | --- | --- |
| **Maternal** | **pH** | **PaCO_2_ (mmHg)** | **Lactate (mmol/L)** | **PaO_2_ (mmHg)** | **Hematocrit (%)** | **SaO_2_ (%)** | **O_2_ Content (mmol/L)** |
| Control | 7.441±0.011 | 35.1±1.3 | 0.67±0.05 | 84.8±0.7 | 27.9±1.1 | 94.2±0.8 | 5.26±0.23 |
| Constant Hyperglycemia | 7.470±0.008* | 36.7±0.5 | 0.68±0.13 | 87.6±2.1 | 25.5±2.1 | 95.9±0.6 | 4.82±0.41 |
| Pulsatile Hyperglycemia | 7.473±0.012* | 33.6±0.7 | 1.01±0.31 | 89.1±2.0 | 31.5±1.2 | 97.2±1.7 | 5.50±0.7 |
| **Fetal** |  |  |  |  |  |  |  |
| *Basal Period* |  |  |  |  |  |  |  |
| Control | 7.348±0.012 | 50.6±0.5 | 1.81±0.21 | 18.7±0.9 | 31.8±1.0 | 50.5±3.9 | 3.14±0.23 |
| Constant Hyperglycemia | 7.329±0.010 | 54.7±0.6* | 3.84±0.92* | 17.0±1.3 | 35.5±2.5 | 42.8±5.6 | 2.85±0.33 |
| Pulsatile Hyperglycemia | 7.376±0.007^#^ | 48.5±0.9^#^ | 1.85±0.19^#^ | 20.7±1.4 | 32.3±1.4 | 48.9±2.6 | 2.92±0.14 |
| *Hyperglycemic Period* |  |  |  |  |  |  |  |
| Control | 7.322±0.012^◊^ | 52.4±0.7^◊^ | 2.25±0.24^◊^ | 18.5±0.9 | 31.4±0.9^◊^ | 47.0±3.6^◊^ | 2.86±0.20^◊^ |
| Constant Hyperglycemia | 7.324±0.008 | 55.8±1.2^◊^* | 3.92±0.76* | 16.7±1.3 | 34.3±2.5^◊^ | 41.3±5.5 | 2.64±0.29^◊^ |
| Pulsatile Hyperglycemia | 7.350±0.007^◊^ | 49.4±0.9^#^ | 2.33±0.21^◊^ | 20.1±1.2 | 31.7±1.3^◊^ | 46.9±2.8 | 2.80±0.18 |
| Fetal Basal and Hyperglycemic periods refer to measurements made immediately before and during the fetal square-wave hyperglycemic clamp, respectively. * indicates a significant difference from Control, ^#^ indicates a significant difference from chronic constant hyperglycemic group, ^◊^ indicates a significant difference between basal and hyperglycemic periods within a treatment group, (P<0.05). | | | | | | | |
|  |  |  |  |  |  |  |  |

**Supplementary Figure. Fetal glucose stimulated insulin secretion.** A square-wave fetal hyperglycemic clamp beginning at time zero was used to test insulin secretion in control (A), CHG (B), and PHG (C) fetuses (glucose concentrations are plotted in the upper panels and insulin concentrations in the lower panels) on day seven. * indicates a significant difference from baseline concentrations (-25, -15, -5 minutes), P=0.029.


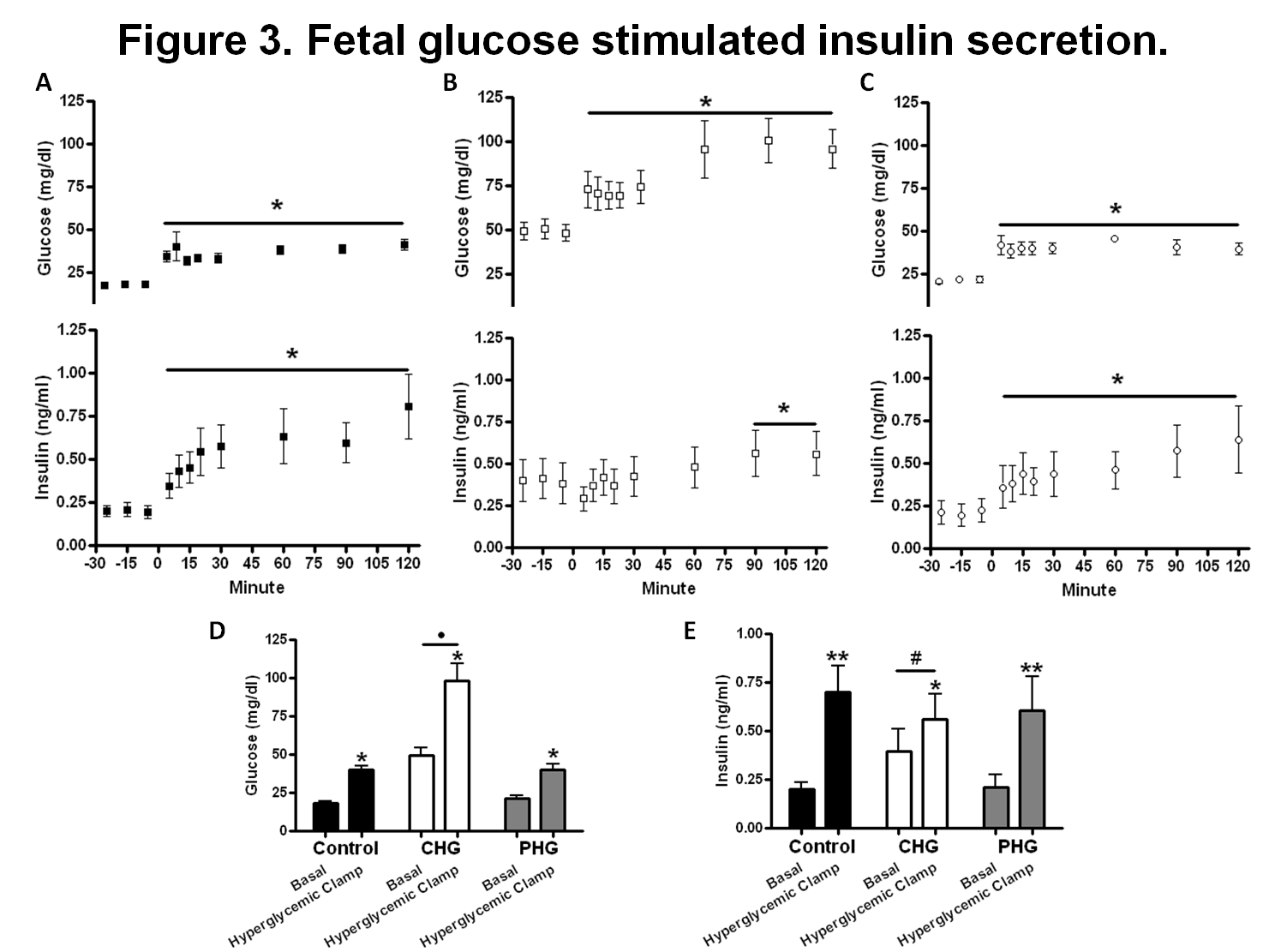

Supplement: Supplementary file 1 — “The supplementary material includes a table with maternal and fetal arterial blood gas and acid base data following the chronic experimental infusions. Also included in this table are the fetal blood gas and acid base data during the fetal hyperglycemic clamp. The supplementary material also includes a figure which shows time specific insulin and glucose concentrations during the fetal hyperglycemic clamp.” [file 812094.f1.docx]
